# The ABC-associated Immunosenescence and Lifestyle Interventions in Autoimmune Disease

**DOI:** 10.2478/rir-2022-0021

**Published:** 2022-10-20

**Authors:** Pinglang Ruan, Susu Wang, Ming Yang, Haijing Wu

**Affiliations:** 1 Department of Dermatology, Second Xiangya Hospital, Central South University, Hunan Key Laboratory of Medical Epigenomics, Changsha, Hunan Province, China; 2 Department of Anesthesiology, Second Xiangya Hospital, Central South University, Changsha, Hunan Province, China

**Keywords:** age-associated B cells, autoimmune disease, immunosenescence, lifestyle interventions

## Abstract

Aging-associated immune changes, termed immunosenescence, occur with impaired robust immune responses. This immune response is closely related to a greater risk of development of autoimmune disease (AID), which results in increased levels of autoantibodies and increased morbidity and mortality. In addition, lifestyle-related risk factors play a pivotal role in AID, which may be probable via senescence-related immune cell subsets. Age-associated B cell (ABC) subsets have been observed in those who have rheumatoid arthritis (RA), systemic lupus erythematosus (SLE), and multiple sclerosis (MS). Here, this review aims to highlight the mechanisms of ABCs with lifestyle interventions in AID, especially how immunosenescence affects the pathogenesis of AID and the future of aging-associated lifestyle interventions in immunosenescence of AID.

## Introduction

Longevity has attracted curiosity and excited attention throughout the history of humankind. In contrast, aging, which is defined as an array of time-dependent functional impaired changes of physiological, epigenomic, metabolic, and immunological alterations, is the first and only key player in regulation of longevity.^[1]^ Immunosenescence is defined as collective abnormal changes of the immune cell with age, which finally affects the disease process directly or indirectly.^[2]^

Recently, numerous studies have demonstrated the importance of age-associated B cell (ABC) subsets in promoting the process of immunosenescence.^[3]^ During aging, ABCs increased in number and then immune responses declined.^[4]^ T cell senescence is the key driver, including the accumulation of dysfunctional, terminally differentiated T cells with abundant proinflammatory factors and abnormal expression of terminal-differentiation markers, which downregulated molecules CD27 and CD28 and upregulated the killer cell lectin-like receptor subfamily G member 1 (KLRG1) and CD57.^[5]^ And ABCs, another key player, have been demonstrated to be in increased levels in autoimmune diseases (AIDs) and affect the morbidity and mortality of those with AID.^[6,7]^ Thus, ABCs have been one of the highlights of immunosenescence. Further, as an antigen-experienced pool, it is important for particular signaling circumstances. ABC-related immune senescence, which includes reduced B cell production and increased systemic and local inflammatory mediators, greatly influenced AID. In addition, lifestyle intervention is an effective and reproducible intervention in AID. A lot of studies show that lifestyle intervention could affect the pathogenesis of AID and the process of immunosenescence.

Herein, we review the generation and differentiation of the ABCs and then recount their effects and development in AID and different lifestyles in detail. Finally, the relationship between lifestyle interventions and anti-senescence effects in AIDs is considered.

## The Generation and Differentiation of ABCs

The ABC subset, a novel B lymphocyte subset, was defined as the senescence-related immune cell subset with damped immune responses. However, for their described criteria, both IgM^+^ CD21^−^ CD35^−^ CD23^−^ B220^+^ CD19^+^ B cells^[8]^ and Cd11c^+^ B220^+^ CD19^+^ B cell^[9]^ were defined as ABCs. Subsequent findings showed that T-bet is particularly important for ABCs.^[10]^ Although there are many distinguishing features of ABCs, they had the same function with the several same key features.

It is undeniable that ABCs were an inevitable outcome during the aging process. Similar to elderly mice, the frequency and number of ABCs of the B cell pool in the elderly were increased.^[7]^ Studies show that, especially in late senescence, half of the total splenic B cells is ABCs.^[6]^ABCs increase because of genetic and epigenetic events accumulating with age. These belong to naturally occurring ABCs that seem to be present in all animals.

But where do these ABCs come from? One can be sure that ABCs can be deeply involved with follicular (FO) B cells. Studies found that FO B cells can differentiate into the ABC after stimulation via Toll-like receptors (TLR) 7 or TLR9 but not after stimulation via B-cell receptor (BCR) or CD40,^[9]^ suggesting that FO B cells could be one of the origins of ABCs. Moreover, ABCs differ from B1 cells. A subsequent study found that the level of T-bet was positively correlated with IL-21 or IFN-γ mediated TLR7 signal, which suggested that TLR-driven activation is the key and important driver of T-bet^+^ B cells.^[11]^ In addition, ABC differentiation is reasoned as co-stimulation of TLR7 and IFN-γ or IL-21.^[6]^ Thus, TLR7 is important and necessary for ABC fate. However, although BCR signals are dispensable for ABC responses, TLR signals with BCR signals can synergistically promote the proliferation and differentiation of ABCs.^[8]^

However, we still need more comprehensive research to get the complete model description with more details to drive B cells to ABCs. As we know, with the co-stimulation of TLR signals and IL-21 or IFN-γ, FO B cells can differentiate into the ABCs. Before that, on the contrary, IL-4 negatively regulated the development of ABCs, which indicated the unknown complex development process of ABCs. T-bet^+^ B cells were not generated with the lack of MHC-II and CD40, suggesting that the germinal center (GC) B cells may be one origin for their generation.^[12]^ Nonetheless, this has not yet been directly demonstrated. But Wang S, *et al*. proposed that CD11c^hi^ T-bet^+^ B cells were differentiated into autoreactive plasma cells (PCs) with IL-21.^[13]^ In addition, Jenks *et al*^[14]^ showed that IgD^−^ CD27^−^ B cell expansions which reflected CD11c^+^ cells are the transitional stage of PCs. These studies show that ABCs with loss of T-bet are key to developing into PCs. Thus, increased levels of autoantibodies with age may be associated with the accumulation of ABCs.

## The ABC Subset in AIDs

Aging is the pivotal impeller in increased incidence of AIDs. Furthermore, ABCs have emerged as a critical driver of AIDs, aging-associated diseases, and infections.^[15]^ Particularly, ABCs in AID fulfill more complicated roles that promote the pathogenesis of complex mechanisms of rheumatoid arthritis (RA), systemic lupus erythematosus (SLE), and multiple sclerosis (MS). Thus, a deeper and broader understanding of ABCs could be important for tailoring successful and effective therapies of targeting these cells in AID.

### The ABC Subset in SLE

SLE is a complex AID with female susceptibility, aberrant activity of the immune system, and a high degree of heterogeneity.^[16,17]^ ABCs are a newly pathogenic B cell subset in SLE,^[18]^ which has attracted extensive attention for its development and function in SLE.

Numerous studies have demonstrated that increased ABCs were observed in lupus mice or SLE patients. A study found that T-bet^+^ CD11c^+^ B cells were positively correlated with the high level of antibodies and meanwhile promoted high anti-chromatin IgG production.^[19]^ Notably, the increased expression of Blimp1 and CD138 in ABCs indicates that it is in the transitional stage before differentiation into PCs, which is also consistent with the previous description that increased levels of autoantibodies with age may be associated with the accumulationofABCs.

In addition, TLR7 is the crucial driver in SLE, which regulates the GC of B cell and the production of autoantibodies.^[20]^ And the TLR7^Y264H^ variant, one TLR7 variant that could enhance TLR7 signaling, drives the increased senescence-associated pheno-type, including the accumulation of ABCs (B220^+^ CD21^–^ CXCR5-CD19^high^ CD11c^+^) and GC B cells (CD19^+^ CD95^+^ BCL6^+^) in SLE.^[21]^ These data suggested that TLR7 is an important upstream driver of MyD88 dependence in human SLE, causing accumulation of ABCs. Notably, authors also proposed that compared with GC B cells, extrafollicular ABCs may be the real culprit of SLE, suggesting that ABCs fulfill a decisive role in SLE.

As mentioned in the previous description, ABC generation is also mediated by IL-21 signaling. Recently, studies show that besides TLR7, ABCs were also regulated by IL-13Rα1-mediated signaling in lupus, which mainly affected the IL-21-mediated signaling.^[22]^ Absence of IL-13Rα1 has negative effects on accumulation of ABCs. Interestingly, levels of IL-13Rα1 in ABC expression are higher than those in FOB cells, showing that upregulated IL-13Rα1 is an another underlying mechanism during which ABCs develop into PCs besides the loss of T-bet. But it is not clear whether there is a relationship between IL-13Rα1 and T-bet.

Zhu *et al.*^[23]^ indicated that IL-10 induces the production of ABCs. It was also found in our previous studies that IL-10 is one risk factor for SLE via upregulating AIM2 expression and then regulating B cell differentiation.^[24]^ To sum up, ABCs are associated with the pathogenesis of SLE and regulated by a variety of mechanisms in SLE.

### The ABC Subset in RA

RA is an AID with systemic and chronic inflammation in joints and increased levels of autoantibodies.^[25]^ RA is significantly associated with immunosenescence and increases with aging, which has been considered a common age-related disease.^[26]^ Increased production of the senescence-associated secretory phenotype (SASP), including TNFα, IL-1β, IL-6, and IFN-γ, has been observed in RA.^[26,27]^ Many studies have reported the mechanism and effects of aging-associated T cells in RA, but there are no well-reported studies on ABCs.

Recently, Qin *et al.*^[28]^ indicated that ABCs were also observed to promote the pathogenesis of RA through affecting tumor necrosis factor-α (TNF-α)-mediated pathways. Meanwhile, they found that increased ABCs were positively promoting disease activity. Increased ABCs interacted with the primary fibroblast-like synoviocytes (FLSs) via TNF-α and promoted inflammation in RA. In addition, a study found that IL-21, IFN-γ, and IL-10 levels were the likely risk factors that affected ABCs in RA patients.^[29]^ Thus, ABCs are associated with the pathogenesis of RA so that when ABCs increase due to internal factors or external circumstances in RA, like latent γ-herpesvirus infection, ABCs could exacerbate arthritis.

Furthermore, although these demonstrate the accumulation of ABCs in RA, the mechanisms underlying generation and differentiation of ABCs are complex in RA. Following in the footsteps of previous studies, more studies are needed in RA.

### The ABC Subset in Other AIDs

MS is a lifelong, chronic neurodegenerative and inflammatory AID. Characteristically, the formation of demyelinating lesions in the central nervous system is associated with immunosenescence in MS.^[30,31]^ Currently, immunosenescence might be involved in MS where some features of immunosenescence were observed, like the accumulation of CD4^+^ CD28^−^ T cells.^[30,32]^ Yet, there are few studies of the aging-associated mechanisms in MS, especially for ABCs. Only one study showed that ABC (CD21^−^ CD11c^+^ B cells) frequencies were increased in MS patients.^[33]^ Moreover, Epstein-Barr virus (EBV), as the risk factor of MS, is deeply associated with MS through molecular mimicry. The latest study indicated that the ABCs (CD19^+^ CD11c^+^ CD21^−^ T-bet^+^) were a key player in the process of EBV-triggered autoimmunity.^[34]^ But whether EBV promotes MS via ABCs as it does in RA is unclear.

Several studies suggest that MS could be controlled by immunosenescence. Therefore, an understanding of immunosenescence in MS is necessary to facilitate targeted therapies.

In summary, ABCs have emerged as critical drivers of AID that need further research to better explore successful and effective therapies for AID.

## Senescence Under Control By Different Lifestyles

Importantly, ABC increases in people at different levels in different regions also vary greatly, which may be attributed to the difference in lifestyle, such as exercise and dietary habits that affect immunosenescence positively or negatively. Thus, correct lifestyle adjustments are necessary, especially for those with age-related diseases.

### Effects of Diets on Senescence

Diets are a basic need which are also the key driver of a healthy life.^[35]^ Diets may directly affect immunosenescence via counteracting the age-related inflammation.^[36]^ In fact, studies have also shown that fasting can effectively reverse immunosenescence in mice.^[37]^ Numerous studies have demonstrated that high intakes of healthy food such as whole grains, vegetables and fruits, nuts, and fish were positively associated with anti-aging effects in health promotion and disease prevention. For example, the Mediterranean dietary pattern (MedDiet), a plant-based common dietary pattern, could greatly promote longevity.^[38]^ Moreover, the intake of essential amino acid tryptophan and n-3 polyunsaturated fatty acids (PUFAs) has revealed that it may affect immune activation responses to adjust health and senescence.^[39]^

Dietary restriction (DR) is another effective method to increase health and counteract immunosenescence.^[40]^ Fontana and Partridge^[41]^ indicated that DR is important for improving health and function. Whether it's intermittent fasting or adjusting circadian eating rhythms, it can promote health and longevity. DR is one of the other effective interventions that can delay aging,^[42]^ which may be associated with reduced expression of IL-1β, IL-6, and TNF-α, as well as change of dysfunctional immune cells. However, Choi *et al*^[40]^ reported that DR may depend directly on the anti-aging effects of its dietary components rather than the method itself. This forces us to understand that what we eat may be just as important as how we eat, especially for health and longevity regulation. One comprehensive review showed the anti-senescence mechanism of nutritional elements such as carbohydrates, proteins, fatty acids, vitamins, minerals, polyphenols, and probiotics.^[43]^

In fact, diet is a major factor that alters the composition and metabolism of the gut microbiome. Caloric restriction first affects the composition of the gut microbiome, which on the one hand improves metabolic health, on the other hand affects T and B cells, and ultimately inhibits immunosenescence.^[44]^ In a recent study, the authors showed that caloric restriction, specifically the very-low-calorie diet (VLCD), can reshape gut microbiota and affect microbial metabolism, reducing the concentration of leucine.^[45]^ The relationship between gut microbiome and senescence has also received extensive attention and discussion.^[46,47]^ Changes in the diversity and composition of the gut microbiota can be affected by age, and dysregulation of the gut microbiota can affect health and longevity.

Especially, in one recent dietary review, Green, C. L., described molecular mechanisms of DR promoting health and longevity in detail: the changed activity of AKT, FOXO, mTOR, nicotinamide adenine dinucleotide (NAD+), AMP-activated protein kinase (AMPK), and fibroblast growth factor 21 (FGF21) is influenced by diet control.^[48]^ Likely, ine one review published in the journal *Cell*, the author indicated the mechanisms of diet, longevity, and disease, including macronutrient composition and levels, fasting, and caloric restriction.^[49]^

In conclusion, one safe, feasible, and effective dietary pattern is beneficial to health and longevity.

### Effects of Exercise on Senescence

Exercise is another healthy lifestyle habit that deserves attention. According to the International Exercise Recommendations in Older Adults (ICFSR), senescence is related to different lifestyles.^[50]^ In contrast, exercise slows down senescence effectively. There is plenty of evidence that exercise can influence immunosenescence to promote longevity,^[51,52]^ such as decreased senescent T cells and increased natural killer (NK) cell cytotoxic activity.

A recent randomized controlled trial showed a reduction in biomarkers of aging after a lifestyle intervention, supporting a strong causal relationship between exercise and aging.^[53]^ In this study, authors indicated that slowing down DNA methylation (DNAm), one of the healthy aging biomarkers, is associated with increasing exercise. Meanwhile, exercise delays T cell senescence via modifying immune cell phenotypes and metabolic status.^[54]^ Padilha *et al*^[55]^ also proposed that exercise can promote health and longevity. Likely, a cross-sectional study found that exercise has a beneficial effect on the suppression of immunosenescence.^[56]^ Interestingly, although the study found that aerobic exercise had few effects on adaptive immune cell,^[57]^ it reduced senescent T cells in older adults,^[58]^ suggesting that exercise possibly specifically affects immunosenescence.

In summary, physical activity and sedentary behavior can affect immunosenescence. However, further research is needed to better understand the function of the exercise for senescence, especially for aging-associated T cells and B cells.

## Lifestyle Interventions and Anti-Senescence Effects in AIDs

Lifestyle-related risk factors play a pivotal role in AID such as RA,^[59]^ SLE^[60]^ and MS,^[61]^ which are necessary to provide correct management and prevention.

The impact of dietary interventions should not be neglected in recent years. Studies have shown that high sodium intake may aggravate RA.^[62]^ In contrast, omega-3 fatty acids seem to have a protective effect against RA,^[63]^ which may be by preventing autoantibody generation in RA.^[64]^ And recent findings suggest that privative diet^[65]^ or MedDiet^[66]^ can better control inflammation in RA patients. Meanwhile, nutrients, fruits, and green vegetables were negatively associated with RA.^[67]^ Although no direct relationship has been established, it is possible that those increase anti-senescence effects to slow down or restrain the pathogenesis of RA.

The National MS Society has increased lifestyle physical activity recommendations for MS, including at least 30 min of activities and so on. A lot of findings suggest the beneficial effects of dietary interventions on MS.^[61,68]^ The mechanisms of lifestyle interventions have not yet been fully elucidated for AID.

A systematic review to assess the effects of lifestyle habits on SLE showed that suitable lifestyle is beneficial to the management of SLE^[69]^ and is a kind of complementary treatment option in SLE patients through foods.^[70]^ And dietary interventions possessed the efficacy of reducing the IL-6 and IL-17 levels in SLE.

However, the mechanism of anti-inflammation and anti-immunosenescence of lifestyle interventions is complex and has not been fully studied in AID. What calls for special attention is that changes in the microbiome, or dysbiosis, are also influenced by lifestyle and dietary patterns.^[71, 69, 72]^ The micro-biome, as a new anti-aging therapeutic strategy, has been proposed to influence the immune system and is a potential determinant of healthy aging.^[47]^ Age-associated microbiome changes could be a potential driver of immunosenescence that require further investigation.

## Conclusions

Immunosenescence, which naturally increases with age, is also associated with external factors such as the environment and disease. ABCs have become a core factor affecting human health by modulating the immune response. As a result, a great deal of attention and research have been focused on age-related interventions that can help prevent and improve AID by reversing aging and increasing beneficial effects. Importantly, aging is strongly associated with autoimmunity, which in turn is causally associated with dietary changes and physical activity. But further research is needed to strengthen this hypothesis, particularly ABC changes among them. In toto, immunosenescence-based therapies are increasingly attractive as new strategies for AID treatment. In addition, the relationship among microbiome dysregulation, aging, and AID has been demonstrated.^[47,73]^ Therefore, it is worthwhile to further study the role of microorganisms as a dietary therapy in AID.

**Figure 1:**
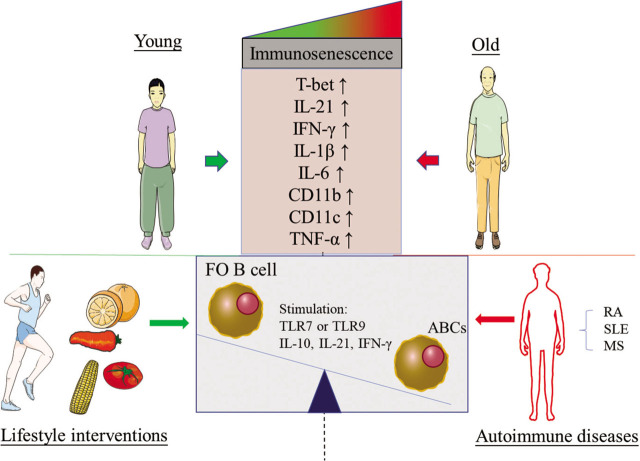
Age-related changes on ABC subset in the health and disease. Compared the young, the frequency and number of ABCs (CD21^−^ CD35^−^ CD23^−^ B220^+^ CD19^+^ B cells and CD19^+^ Cd11c^+^ T-bet^+^ B cell) in the elderly were increased. Similarly, AIDs, including SLE, RA and MS, can contribute to the increase of ABCs. Increased production of TNF-α, IL-1β, IL-6, IL-21 and IFN-γ has been observed with immunosenescence in the old and patients with AIDs. In addition, lifestyle interventions had the beneficial effects on the suppression of immunosenescence. Green arrow: negative regulation; Red arrow: positive regulation; Figure created with Adobe Photoshop CC 2019. ABCs, age-associated B cell; AIDs, autoimmune diseases; FO, follicular; MS, multiple sclerosis; RA, rheumatoid arthritis; SLE, systemic lupus erythematosus.
